# Microwave and Terahertz Properties of Spark-Plasma-Sintered Zr_0.8_Sn_0.2_TiO_4_ Ceramics

**DOI:** 10.3390/ma15031258

**Published:** 2022-02-08

**Authors:** Liviu Nedelcu, Mihail Burdusel, Mihai Alexandru Grigoroscuta, Cezar Dragos Geambasu, Monica Enculescu, Petre Badica, Marian Gabriel Banciu

**Affiliations:** National Institute of Materials Physics, Atomistilor 405A, 077125 Magurele, Romania; mihai.burdusel@infim.ro (M.B.); mihai.grigoroscuta@infim.ro (M.A.G.); cezar.geambasu@infim.ro (C.D.G.); mdatcu@infim.ro (M.E.); petre.badica@infim.ro (P.B.)

**Keywords:** ceramics, zirconium tin titanate, Zr_0.8_Sn_0.2_TiO_4_, spark plasma sintering, microwave dielectric properties, terahertz time-domain spectroscopy

## Abstract

Zr_0.8_Sn_0.2_TiO_3_ (ZST) powders synthesized by solid-state reaction were subject to processing by spark plasma sintering (SPS). A single-phase ceramic with a high relative density of 95.7% and 99.6% was obtained for sintering temperatures of 1150 °C and 1200 °C, respectively, and for a dwell time of 3 min. In order to reduce the oxygen vacancies, as-sintered discs were annealed in air at 1000 °C. The dielectric loss of the annealed samples, expressed by the *Q* × *f* product, measured in the microwave (MW) domain, varied between 35 THz and 50 THz. The intrinsic losses (*Q* × *f* ~ 60 THz) were derived by using terahertz time-domain spectroscopy (THz-TDS).

## 1. Introduction

The rapid development of wireless communications and information technology benefits from MW dielectrics. The high dielectric constant and low loss at very high frequencies allow the development of equipment with reduced size and improved characteristics. MW dielectrics are essential for the fabrication of dielectric resonator (DR) stabilized oscillators, high-selectivity filters and multiplexers, dielectric resonator antennas, metamaterials, etc. [1,2,3,4,5,6,7,8]. Several MW dielectrics have been reported with attractive properties for applications [9,10,11,12,13,14,15], but some technological limitations are encountered when production at a large scale is attempted. Ceramic MW dielectrics allow scalability for extensive manufacturing and show cost-effectiveness; therefore, they are MW dielectrics used most for production of MW passive devices.

During the last decades, ZST ceramics have been extensively investigated as low-loss MW materials for substrates and dielectric resonator (DR) applications [11,16,17,18,19,20,21]. ZST ceramics are materials that are difficult to sinter. For high-quality ceramics, high densities are required. To overcome this problem, additives are often introduced, but having a large amount of them degrades the dielectric properties [11]. Another approach, in combination with additives or not, is to use special processing technologies. Pressure-assisted methods are popular; among them, SPS shows useful specific features. SPS applies a uniaxial pressure on a die system loaded with the powder to be sintered. Heating is carried out by passing an electrical current through the die and/or the sample. It is considered that the pulsed component of the applied current promotes the activation of unconventional effects (such as hot-spot formation, grain boundary cleaning, electrodiffusion, the occurrence of sparks and plasma states, and so on [22,23,24,25]) that speed up sintering and decrease the processing temperature. SPS is also flexible, allowing high heating and cooling rates. All the presented features promote SPS as a valuable technology that can preserve the particle size and phase composition. Moreover, by creating various shapes [26], it is possible to tailor the functionality of the sintered materials. Over time, SPS has been successfully used to sinter various materials [22,23,24,25,26], including MW dielectrics [27,28,29,30]. However, no study regarding the MW dielectric properties of spark-plasma-sintered ZST materials has yet been reported. Articles are available on microstructural observations, low-frequency dielectric properties [31] and THz absorption [32]. Considering these results as a reference point, one may expect that ZST produced by SPS will have sufficiently low absorption.

In this work, to the best of our knowledge, the MW properties of ZST ceramics fabricated by SPS are presented for the first time. High values of the *Q* × *f* product (*Q* is the inverse of the dielectric loss tangent and *f* is the measurement frequency) from MW to THz were achieved for ZST DRs fabricated by conventional sintering [18,33]. These promising results motivated the current study, namely fabrication by SPS and characterization of high-density ZST with the aim of low dielectric loss, and a comparative analysis between the dielectric MW and THz properties. The intrinsic dielectric loss was assessed from the THz data.

## 2. Materials and Methods

### 2.1. Samples Preparation

The ZST powder was prepared by a solid-state reaction from the raw materials ZrO_2_ (Alfa Aesar, 99.5% purity, Kalrsruhe, Germany), SnO_2_ (Alfa Aesar, 99.9% purity, Kandel, Germany) and TiO_2_ (Sigma-Aldrich, 99.8% purity, Steinheim, Germany). To reduce the synthesis temperature, 2 wt.% La_2_O_3_ (Sigma-Aldrich, 99.9% purity, Steinheim, Germany) and 1 wt.% ZnO (Sigma-Aldrich, 99.0% purity, Steinheim, Germany) were added. The oxides were homogenized in deionized water for 6 h at 400 rpm in a PM200 planetary mill (Retsch, Haan, Germany) using zirconia balls and jars, and subsequently calcined in air at 1150 °C for 2 h.

**Figure 1:**
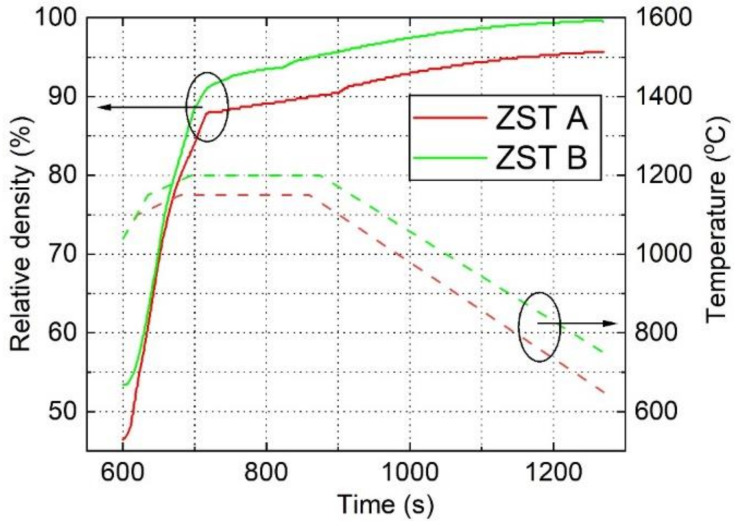
Relative density during SPS processing and the designed heating temperature versus SPS processing time for the samples ZST A (green lines) and ZST B (red lines).

The calcined powder (10 g for each sample) was loaded into a graphite die with a inner diameter of 20 mm. The die was placed in the HP D5 sintering furnace (FCT Systeme, Rauenstein, Germany). The initial vacuum in the chamber was 40 Pa. The maximum uniaxial pressure applied on the samples during SPS was 90 MPa. The heating rate was about 200 °C/min. Sintering temperatures of 1150 and 1200 °C were maintained for 3 min (Figure 1), and the cooling rate was about 70 °C/min. The variation of the relative density of the samples during SPS with time and temperature is presented in Figure 1. Relative density was estimated on the basis of the bulk density of the final sintered product measured by the Archimedes method (see Section 2.2) and on displacement curves of the pistons measured in situ during processing. The methodology for relative density estimation is described in [34].

The as-sintered samples were polished to remove the superficial region contaminated with carbon and were subsequently annealed in air at 1000 °C for 10 h to reduce the oxygen vacancies. For MW and THz measurements, lamellas of about 10 mm × 10 mm × 0.3 mm were cut from annealed discs with a SYJ-150 low-speed diamond saw (MTI Corporation, Richmond, VA, USA).

### 2.2. Samples Characterization

The bulk density of the ZST discs was measured by the Archimedes method with a Density Determination Kit (YDK01MS) mounted on a CUBIS MSA224S analytical balance (Sartorius Lab Instruments, Goettingen, Germany).

The crystalline structure of ZST discs was examined by X-ray diffraction (XRD) using a Bruker–D8 Advance diffractometer (Bruker AXS, Karlsruhe, Germany) in a Bragg–Brentano configuration. Measurements were performed from 20° to 70°, with 2θ-steps of 0.02° and 2 s counting time. A Ni-filtered copper $K_{\alpha 1}-K_{\alpha 2}$ radiation doublet and a LynxEye one-dimensional detector were used for recording the XRD patterns; an Al_2_O_3_ reference material (NIST SRM 1976) was used for 2θ calibration of the diffractometer.

The morphology of the fractured samples was investigated with a Zeiss Evo 50 XVP Scanning Electron Microscope (Carl Zeiss, Oberkochen, Germany), working in High Vacuum mode. The microscope was equipped with a LaB_6_ filament and a SE2 detector. Images were obtained for an acceleration voltage of 20 kV.

The THz-TDS measurements were carried out on ZST lamellas with an IRS 2000 PRO spectrometer (Aispec, Tokyo, Japan) in transmission set-up. The atmospheric vapor absorption needed to be reduced. To achieve this requirement, the sample chamber was vacuumed below 10 Pa with a ACP 40 pump (Pfeiffer Vacuum Technology, Asslar, Germany). The absorption coefficient, relative permittivity ($\varepsilon_{r}$) and dielectric loss tangent (tanδ) were extracted from the time-domain data by using TeraLyzer commercial software (Menlo Systems, Martinsried, Germany) and subsequently verified at several frequencies by an amplitude-based procedure detailed elsewhere [35].

The ZST lamellas were characterized in MW with the Split Post Dielectric Resonator (SPDR) technique [36]. The SPDR (QWED, Warsaw, Poland) was connected to an E8361A Vector Network Analyzer (Agilent, Santa Clara, CA, USA), and $\varepsilon_{r}$ and tanδ were determined from transmission (*S*_21_ parameter).

## 3. Results and Discussion

### 3.1. Density, Structural and Microstructural Details of the ZST Ceramics

Following the sintering, polishing and annealing processes, ZST discs with a relative density higher than 95.7% of the theoretical density were obtained. The bulk density of the ZST samples versus sintering temperature is shown in Table 1. The ZST B sample sintered at a higher temperature of 1200 °C exhibited a high bulk density of 99.6%, a result that can be considered an excellent achievement.

**Figure 2:**
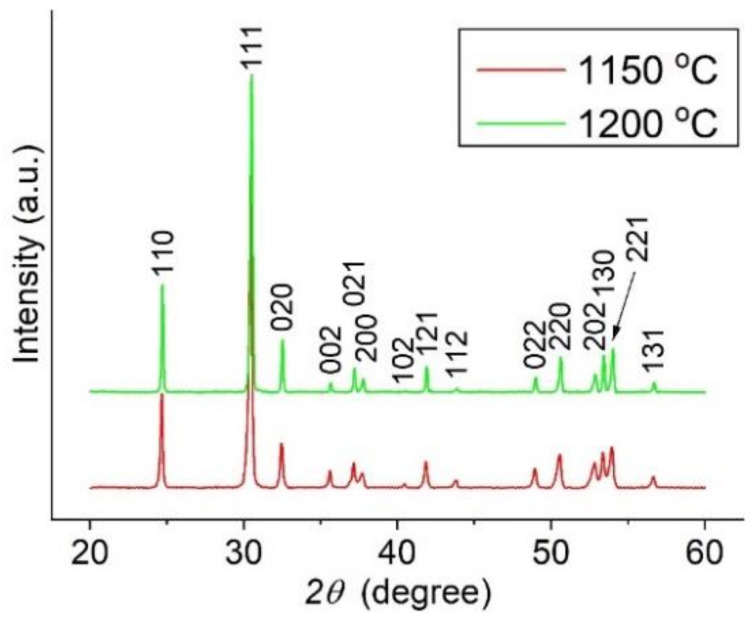
XRD patterns for annealed ZST Samples A and B (Table 1) obtained for sintering temperatures of 1150 °C and 1200 °C, respectively.

The structural XRD data for Samples A and B (Table 1) after annealing were examined using Bruker Diffrac Plus Basic Package Evaluation v.4.2.1 (Bruker AXS, Karlsruhe, Germany) and phase identification was conducted with the ICDD PDF4+ 2021 database (International Centre for Diffraction Data, Newtown, PA, USA). The XRD patterns (Figure 2) show the formation of the ZST (αPbO_2_-type structure) with a small (110) preferential orientation. For both sintering temperatures, the diffraction lines were indexed with respect to the *Pnab* space group (ICDD 01-081-2214 file). Within the detection limit of the equipment, no secondary phases were detected. The lattice constants of the orthorhombic unit cell are listed in Table 1. As can be seen, the sintering conditions had a small influence on the lattice constants.

**Figure 3:**
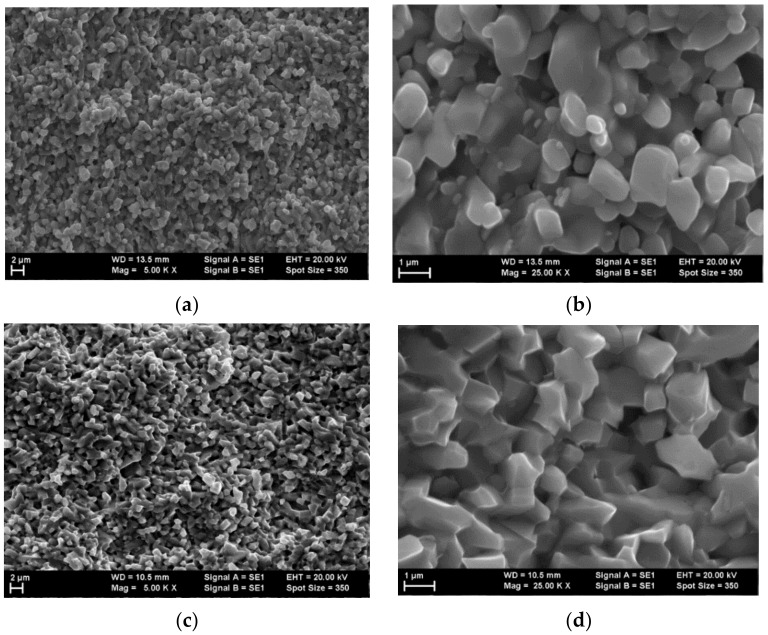
SEM micrographs of the ZST samples sintered at (**a**,**b**) 1150 °C and (**c**,**d**) 1200 °C. The images were recorded at (**a**,**c**) 5000× magnification and (**b**,**d**) 25,000× magnification using an accelerating voltage (EHT) of 20 kV.

The morphology of the studied samples was assessed from SEM images taken on fresh fracture surfaces. Images at different magnifications are presented in Figure 3. From the lower magnification SEM images, it can be observed that the grains have a relatively narrow distribution of dimensions for both types of sample (Figure 3a,c). Some close pores are available in Sample A sintered at 1150 °C with a lower density (Table 1), and they can be observed in the images recorded at high magnification (Figure 3b). The high-magnification observations also revealed a few other differences between the samples. Thus, Sample A sintered at 1150 °C (Figure 3b) contained a fraction of smaller grains mixed with the average-sized grains. The average-sized grains were sintered aggregates of smaller grains. By comparison, in Sample B sintered at 1200 °C (Figure 3d), the grains were uniform and had a narrower distribution of dimensions. At the same time, the edges of the grains of the sample sintered at a higher temperature were sharper compared with the edges from the sample sintered at lower temperature, suggesting better crystal quality.

### 3.2. Dielectric Properties of ZST Ceramics

**Figure 4:**
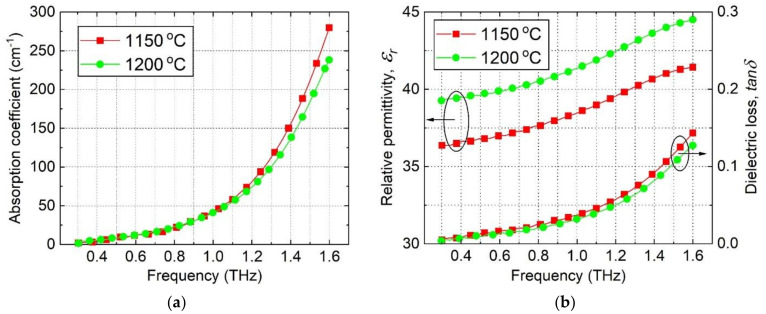
Frequency dependence of (**a**) the absorption coefficient, and (**b**) relative permittivity and the dielectric loss tangent versus sintering temperature for ZST ceramics (Samples A and B were sintered at 1150 °C and 1200 °C, respectively).

In Figure 4, the frequency dependence of the THz absorption coefficient (Figure 4a), the relative permittivity $\varepsilon_{r}$ (Figure 4b) and dielectric loss tangent tanδ (Figure 4b) for the ZST lamellas are depicted. Despite the fact that the instrument’s characteristics allowed measurements on intrinsic silicon up to 7 THz [37], the measurements on ZST were limited to 1.6 THz due to the absorption behavior of ZST. The data plotted for the ZST Sample B showed lower THz absorption and dielectric tangent loss and higher relative permittivity $\varepsilon_{r}$ than Sample A in the entire frequency range.

The results of the MW measurements conducted on ZST lamellas around 16 GHz are shown in Table 2. It can be observed that $\varepsilon_{r}$ slightly increased with an increase in the sintering temperature. This dependence of the permittivity was in agreement with the increase in the bulk density and the better morphology due to the increase in the sintering temperature (Table 1, Figure 3). On the other hand, tanδ showed a larger variation, from 4.6 × 10^−4^ at 1150 °C to 3.2 × 10^−4^ at 1200 °C. As shown in Table 1, the variation of density (or porosity) with sintering temperature was quite low, from 95.7% in Sample A to 99.6% in Sample B. It can be considered that the losses due to the pores should not vary significantly. Therefore, other extrinsic contributions are responsible for increased tanδ of ZST Sample A. For easy comparison, the values for $\varepsilon_{r}$ and tanδ at 0.4 THz are gathered in Table 2.

In the case of a material without defects, the multiphonon absorption theory predicts a constant value for the *Q* × *f* product [38] over a wide frequency range. Due to the increase in tanδ and the decrease in *Q* with an increase in the frequency, the *Q* × *f* product is used more frequently than the tanδ or *Q* in order to describe the dielectric loss of a material. However, in practical cases, due to the presence of the losses induced by defects, the *Q* × *f* product increases with an increase in the frequency [9,11,33,39] and reaches a steady level in millimeter waves (MMWs) [40], which corresponds to the intrinsic losses. We may conclude that, at very high frequencies, the extrinsic losses become negligible and the material exhibits mainly intrinsic losses.

The theory predicts a constant value for $\varepsilon_{r}$ along a very wide frequency band [9,38]. In practice, for the investigated ZST samples, the permittivity data given in Table 2 exhibit only a very small increase from the MW to THz domains.

In addition, the data shown in Table 2 indicate a significant increase in the *Q* × *f* product when the measurement frequency increases from MW to THz for both ZST A and ZST B samples. This dependence proves that the extrinsic losses are reduced in the MMW and THz regions. The intrinsic losses (*Q* × *f* ~ 60 THz) of the ZST samples, which were derived by using THz-TDS, are in good agreement with the data reported previously [9,33,38].

As was stated in Section 1 (Introduction), no data about the MW dielectric properties of the ZST ceramics fabricated by SPS have been reported in the literature. For comparison, Table 3 presents the data reported in the literature for ZST ceramics fabricated by various processing technologies and with different additives. One can observe that the values of $\varepsilon_{r}$ and the *Q* × *f* product of our ZST Sample B are comparable with the best data available in the literature.

Despite the advantages presented above, many oxygen vacancies are formed in oxide materials during the SPS process. This extrinsic factor increases the measured absorption (extrinsic + intrinsic) in dielectric materials, but this shortcoming can be overcome by proper ex situ annealing treatments in air [32]. As can be seen in Table 3, the ZST material proposed in this work exhibits attractive features for high-frequency applications and it was sintered at the lowest temperature.

The high values of the *Q* × *f* product achieved for ZST ceramics recommend SPS for the densification of other already tested powders or of new ones. From a general point of view, the versatility of SPS can be exploited, and we expect to fabricate MW dielectrics in the future with the complex shapes that are needed for the development of passive devices with new and enhanced capabilities.

## 4. Conclusions

SPS has been successfully used to fabricate 99.6% densified single-phase ceramics at 1200 °C/3 min from ZST powders that are difficult to sinter. The oxygen vacancies generated during the processing of oxide powders by SPS were reduced by post-annealing in the air atmosphere at 1000 °C. The dielectric measurements confirmed that the extrinsic factor contributed predominantly to the MW losses; therefore, the intrinsic losses (*Q* × *f* ~ 60 THz) were assessed in the THz data. The ZST Sample B sintered at 1200 °C exhibited a bulk density of 5.17 g/cm^3^, a relative dielectric permittivity $\varepsilon_{r}$ of 38.9 at 16 GHz and 39.3 at 0.4 THz, and a *Q* × *f* product of 50 THz at 16 GHz and 60 THz at 0.4 THz. All the abovementioned parameters of ZST Sample B presented higher values than for ZST Sample A sintered at 1150 °C. The MW dielectric parameters achieved are comparable with the best reported results, which emphasizes the high potential of the SPS technique for the fabrication of low-loss MW dielectrics with extended functional properties.

## Figures and Tables

**Table 1:** The bulk density and lattice constants of ZST ceramics versus the sintering temperature.

Sample	Sintering Temperature (°C)	Bulk Density ^1^ (g/cm^3^)	Lattice Constants		
			*a* (nm)	*b* (nm)	*c* (nm)
ZST A	1150	4.97	0.4771	0.5512	0.5036
ZST B	1200	5.17	0.4764	0.5505	0.5034

^1^ The X-ray density of ZST is 5.193 g/cm^3^ (ICDD file No. 01-081-2214).

**Table 2:** Relative permittivity ($\varepsilon_{r}$), dielectric loss (tanδ ) and *Q* × *f* product of ZST samples measured in the microwave (@ 16 GHz) and terahertz (@ 0.4 THz) domains.

Sample	*ε_r_* @ 16 GHz	*tanδ* @ 16 GHz	*Q* × *f* @ 16 GHz (THz)	*ε_r_* @ 0.4 THz	*tanδ* @ 0.4 THz	*Q* × *f* @ 0.4 THz (THz)
ZST A	36.1	4.6 × 10^−4^	35	36.4	7.3 × 10^−3^	55
ZST B	38.9	3.2 × 10^−4^	50	39.3	6.7 × 10^−3^	60

**Table 3:** MW dielectric properties of ZST ceramics in the literature compared with those reported here.

Reference	Powder Synthesis Method	Sintering Temperature (°C)	Additives	*ε_r_*	***Q* × *f*** (**THz**)
[16]	CMO	1360	Fe_2_O_3_, ZnO, NiO	38	39
[17]	CMO	1380	Ta_2_O_5_, ZnO, NiO	38	60
[41]	MA	1600	-	40.3	50
[42]	CP	1325	La_2_O_3_, ZnO	37.6	54
[43]	HT	1280	-	37.5	25
[44]	SG	1300	ZnO	38	55
[45]	FG	1400	-	38.2	57
[33]	CMO	1300	La_2_O_3_, ZnO	37.3	50
This work (sample B)	CMO	1200	La_2_O_3_, ZnO	38.9	50

CMO—conventional mixed oxide; MA—metal alkoxide; CP—co-precipitation; HT—hydrothermal; SG—sol-gel; FG—freeze granulation.

## Data Availability

The data presented in this study are available on request from the corresponding author.
